# GDNF Enhances Therapeutic Efficiency of Neural Stem Cells-Based Therapy in Chronic Experimental Allergic Encephalomyelitis in Rat

**DOI:** 10.1155/2016/1431349

**Published:** 2016-04-26

**Authors:** Xiaoqing Gao, Li Deng, Yun Wang, Ling Yin, Chaoxian Yang, Jie Du, Qionglan Yuan

**Affiliations:** ^1^Department of Anatomy and Neurobiology, Southwest Medical University, Zhongshan Road, Luzhou, Sichuan 646000, China; ^2^Preclinical Medicine Research Center, Southwest Medical University, Zhongshan Road, Luzhou, Sichuan 646000, China; ^3^Department of Anatomy and Neurobiology, Tongji University School of Medicine, Siping Road, Shanghai 200092, China

## Abstract

Multiple sclerosis (MS) is an autoimmune disease in the CNS. The current immunomodulating drugs for MS do not effectively prevent the progressive neurological decline. Neural stem cells (NSCs) transplantation has been proven to promote repair and functional recovery of experimental allergic encephalomyelitis (EAE) animal model for MS, and glial cell line-derived neurotrophic factor (GDNF) has also been found to have capability of promoting axonal regeneration and remyelination of regenerating axons. In the present study, to assess whether GDNF would enhance therapeutic effect of NSCs for EAE, GDNF gene-modified NSCs (GDNF/NSCs) and native NSCs were transplanted into each lateral ventricle of rats at 10 days and rats were sacrificed at 60 days after EAE immunization. We found that NSCs significantly reduced the clinical signs, and GDNF gene-modification further promoted functional recovery. GDNF/NSCs more profoundly suppressed brain inflammation and improved density of myelin compared with NSCs. The survival of GDNF/NSCs was significantly higher than that of transplanted NSCs. Transplanted GDNF/NSCs, in contrast to NSCs, differentiated into more neurons and oligodendrocytes. Moreover, the mRNA expression of oligodendrocyte lineage cells in rats with GDNF/NSCs was significantly increased compared to rats with NSCs. These results suggest that GDNF enhances therapeutic efficiency of NSCs-based therapy for EAE.

## 1. Introduction

Experimental allergic encephalomyelitis (EAE), most commonly used animal model of multiple sclerosis (MS), is an autoimmune disease in the central nervous system (CNS) induced and mediated by myelin-reactive T cells responses against myelin antigens [1]. In EAE and MS, autoreactive T cells migrate from peripheral tissues into the CNS where they are reactivated, thereby triggering an inflammatory cascade that results in extensive loss of myelin and myelinating cells (oligodendrocytes) as well as damage to axons and neurons [2, 3]. Since neural stem cells (NSCs) have abilities to differentiate into various neural cell types [4] and have myelinogenic potential [5] in the injured areas, they may be a potential cell type of replacement therapy for EAE. However, lower survival rate [6] and low levels of differentiation of oligodendrocytes [7] and neurons [8] after transplantation limit the therapeutic efficacy of NSCs.

Glial cell line-derived neurotrophic factor (GDNF), a potent neurotrophic factor, has been demonstrated to have neuroprotection against a variety of neuronal insults [9, 10]. Moreover, GDNF can promote axon regeneration and myelination after spinal cord injury [11–13]. Our previous studies have shown that GDNF gene-modified NSCs provided more efficient neuroprotection for rats subjected to stroke than native NSCs [14]. Therefore, here, we speculate that GDNF may enhance therapeutic efficiency of NSCs therapy for EAE. The purpose of this study is to investigate whether GDNF gene-modified NSCs provide a more efficacious treatment for EAE than NSCs alone.

## 2. Materials and Methods

### 2.1. Culture and Infection of NSCs

The culture and infection of NSCs were performed as described in previous study [14]. Briefly, the cell suspensions from the cerebral hemispheres of newborn Wistar rats (inbred strain, Animal House Center, Southwest Medical University, Sichuan, China) were placed in 25 mL flask at a density of 1 × 10^5^ cells/mL in serum-free DMEM/F12 medium supplemented with 20 ng/mL of epidermal growth factor (EGF), 20 ng/mL of basic fibroblast growth factor (bFGF), and 1% N2 supplement (all from Gibco, USA). After 5–7 days of culture, neurospheres were formed. To obtain GDNF gene-modified NSCs (GDNF/NSCs), neurospheres were infected using GDNF recombinant adenovirus (pAdEasy-1-pAdTrack CMV-GDNF) for 2 days. Adeasy-1 plasmid contains the gene for green fluorescent protein and the titers of viral were 1 × 10^9^ PFU/mL. The expression of GDNF in NSCs was examined by a reverse transcriptase-PCR assay 2 days after infection. The primer sequences are as follows: sense, 5′-CCGAAGATTATCCTGACC-3′, and antisense, 5′-GTAGCCCAAACCCAAGT-3′, and the product length is 242 bp fragment. For labeling the grafted cells* in vivo*, NSCs and GDNF/NSCs were pretreated for 3 days before grafting with 10 *μ*M of 5-bromo-2′-deoxyuridine (BrdU, Sigma, USA).

### 2.2. Neural Differentiation of Neurospheres* In Vitro*

To induced NSCs and GDNF/NSCs differentiation, neurospheres were adhered to coverslips precoated with poly-L-lysine (10 *μ*g/mL, Sigma) in six-well plates in DMEM/F12 medium in the absence of bFGF, EGF, and N2 supplement but with 10% fetal bovine serum (FBS) for 7 days. The cultures were fixed with 4% paraformaldehyde for 30 min and incubated with neuronal specific markers microtubule-associated protein 2 (MAP2, 1 : 100, rabbit, Abcam, UK), astrocytes specific markers glial fibrillary acidic protein (GFAP, 1 : 100, rabbit, Abcam), and oligodendrocyte specific marker galactocerebroside (GalC, 1 : 50, rabbit, Chemicon, USA) overnight at 4°C, followed by appropriate biotinylated secondary antibodies (1 : 100, Wuhan Boster Biological Technology, China) and horseradish peroxidase- (HRP-) streptavidin (Boster) for 30 min at 37°C. Immunoreactivity was visualized with diaminobenzidine (DAB, Boster). Counterstaining by hematoxylin was also performed. A negative control was performed using the same procedures without primary antibody. The ratio of MAP2^+^, GalC^+^, or GFAP^+^ cells to total cells was quantified in 5 regions in 3 coverslips per group.

### 2.3. EAE Induction and NSCs Transplantation

All animal use and relevant experiments were approved by the Chinese Academy of Sciences, PR China. EAE is induced in female Wistar rats by guinea pig spinal cord homogenate emulsified with complete Freund's adjuvant (CFA, Sigma). Briefly, Wistar rats (6–8 weeks old, female, Southwest Medical University) were injected in four footpads with 100 mg of guinea pig spinal cord tissue in 0.4 mL of PBS emulsified with equal volume of CFA supplemented with 6 mg/mL of* Mycobacterium tuberculosis* H37Ra (Shijiazhuang Weitian Scientific Instruments Equipment Co., Ltd., China). On the day of immunization and on day 2, 300 ng of bordetella pertussis toxin (Weitian) in 0.1 mL PBS was injected subcutaneously. Rats were daily monitored for the severity of clinical disease. After immunization, the following scale for clinical symptoms was utilized: 0, no clinical symptom, 1, limpness in tail, 2, hind-leg ataxia, 3, hind-leg paralysis, 4, paraplegia, and 5, moribund or dead. At ten days after EAE induction, rats were anesthetized with intraperitoneal injection of pentobarbital sodium (30 mg/kg) and then were fixed in a stereotactic device (Angle Two*™* Stereotaxic Instrument w/Rat Atlas Product: # 464601, USA). Quantities of 5 × 10^5^ NSCs or GDNF/NSCs in a volume of 10 *μ*L were injected once into each lateral ventricle (bregma as origin, AP = −0.8 to 1.0 mm, L or R = −1.8 to 2.0 mm, and V = −4.0 to 5.0 mm). The control group experienced the same injection with 10 *μ*L saline into each lateral ventricle.

### 2.4. Histological and Immunohistochemical Assessment

Animals were sacrificed with a lethal dose of pentobarbital sodium on day 60 after EAE induction. Fifteen rats (5 per group) were perfused by 4% paraformaldehyde for histopathological analysis. Histopathology and immunohistochemistry were assessed in 6 *μ*m thick paraffin sections at various levels (for bregma −0.2 to −1.6 mm). Hematoxylin-eosin (HE) staining was used to evaluate inflammatory infiltration. 10 nuclei or more gathered in cerebrum white matter or surrounded by a blood vessel were considered as an infiltration lesion. To estimate the degree of inflammation, the number of infiltrates and the number of cells per infiltrate were counted. Luxol fast blue (LFB, Sigma) staining was used to visualize myelin sheath and the measurement parameter was the integrated optical density (IOD).

BrdU immunohistochemistry staining was used to identify grafted cells in brain. Sections were incubated with mouse anti-BrdU (1 : 200, Abcam, UK) at 4°C overnight; then goat anti-mouse IgG (1 : 100, Wuhan Boster Biological Technology, China) was added at 37°C for 30 min. After alkaline phosphatase- (AP-) streptavidin (Boster) was incubated at 37°C for 30 min, 5-Bromo-4-Chloro-3-Indolyl Phosphate/Nitroblue Tetrazolium Chloride (BCIP/NBT, Boster) was used as a chromogen for 10 min. For double-labeling experiments, MAP2, GFAP, and GalC were used to identify differentiated cells from grafted cells. Sections were incubated again with rabbit anti-MAP2 (1 : 100), GFAP (1 : 100), and GalC (1 : 50) at 4°C overnight, and goat anti-rabbit IgG (1 : 100, Boster) was then incubated for 30 min. Horseradish peroxidase- (HRP-) streptavidin (Boster) was incubated for 30 min, followed by diaminobenzidine (DAB, Boster) or 3-amino-9-ethylcarbazole (AEC, Boster) for 10 min as a chromogen. Immunohistochemistry controls were routinely performed with incubations in which primary antibodies were omitted.

Myelin sheath was assessed as IOD in corpus callosum in 3 sections per animal by ImageJ 1.44p software. The infiltrates in the corpus striatum were counted in sections per rats. And 3 fields/striatum were imaged, so 6 fields in bilateral striatum were imaged and counted. Immunopositive cells were counted in 6 fields situated within bilaterally striatum in 3 sections per animal. Measurement was made in a predefined field (0.6 mm × 0.6 mm) by image-pro plus 6.0 software.

### 2.5. Reverse Transcription- (RT-) PCR Analysis

For RT-PCR experiments, fifteen rats (5 per group) were analyzed. Total RNA was prepared from brain tissue at 0.2–1.6 mm behind bregma using TRIZOL reagent (Invitrogen, USA) following the manufacturer's instructions. Two *μ*g of RNA was reversely transcribed into cDNA using a Takara RNA PCR kit (Dalian Takara Biotechnology, China). The reaction conditions were 30°C for 10 min, 42°C for 30 min, 99°C for 5 min, and 5°C for 5 min. Oligonucleotides used as specific primers were as follows: platelet-derived growth factor *α* receptor (PDGF*α*R), sense, 5′-CCAAATACTCCGACATCC-3′, antisense, 5′-CCAGAGCAGAACGCCATA-3′, 404 bp fragment; GalC, sense, 5′-CGGTGCCCTTGTTGTTGTG-3′, antisense, 5′-TGCCGTCTGTTGTTTGTCC-3′, 252 bp fragment; myelin basic protein (MBP), sense, 5′-TTCTTTAGCGGTGACAGGG-3′, antisense, 5′-GGAGCCGTAGTGGGTAGTT-3′, 156 bp fragment; and GAPDH, sense, 5′-ACCACAGTCCATGCCATCAC-3′, antisense, 5′-TCCACCACCCTGTTGCTGTA-3′, 450 bp fragment. cDNA was amplified by 30 cycles and the parameters of amplification were as follows: denaturing for 30 sec, 94°C; annealing for 30 sec, PDGF*α*R at 57°C, GalC at 59°C, MBP at 55°C, and GAPDH at 57°C; extension for 4 min, at 72°C. GAPDH was used as an endogenous control. PCR products were electrophoresed on agarose gels and stained by ethidium bromide.

### 2.6. Statistical Analysis

All measured values were expressed as mean ± SD, and statistical analysis was performed using SPSS software, version 17.0. For the clinical scores, the multiple comparisons were done using two-way repeated measures ANOVA followed by Tukey's posttest for multiple pairwise examinations. For the other histological, immunostaining, and PCR analyses, multiple comparisons were done using one-way ANOVA followed by Tukey's* post hoc* test for multiple pairwise examinations. Difference was considered significant at *P* < 0.05.

## 3. Results

### 3.1. GDNF Promoted Neuronal Differentiation of NSCs* In Vitro*

**Figure 1:**
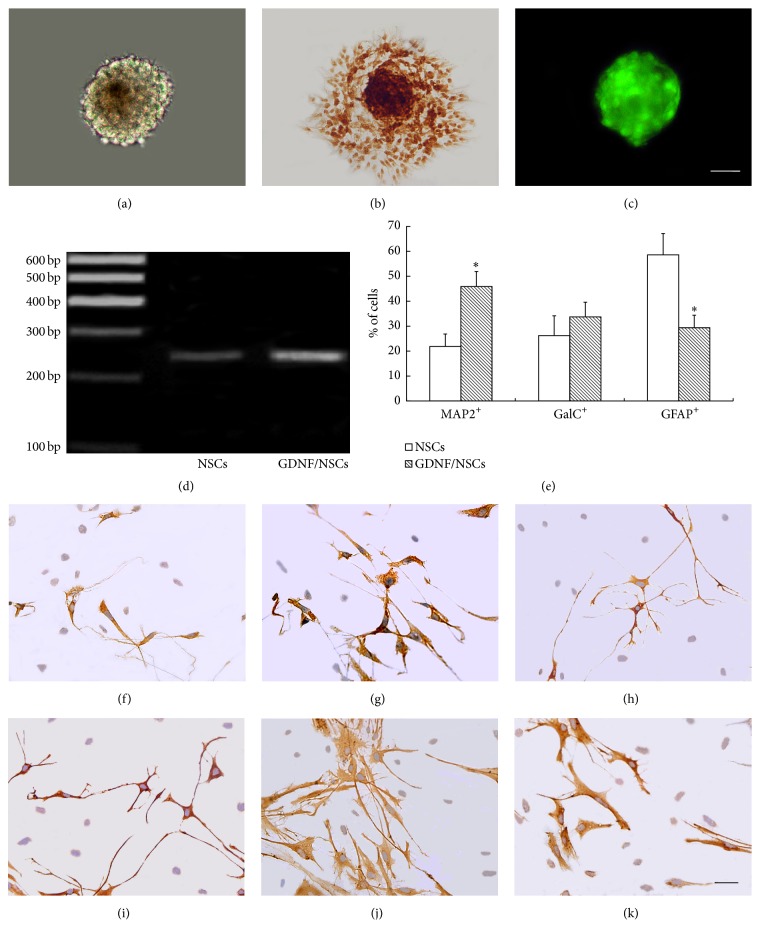
The generation and characterization of the neurosphere* in vitro*. (a) Cultured neurospheres derived from cerebral tissue of newborn rats at culture of 7 d. (b) Immunocytochemical staining of nestin in neurospheres. (c) Neurospheres infected by GDNF recombinant adenovirus for 2 days showed green fluorescence under fluorescence microscope. (d) RT-PCR showed higher level of GDNF mRNA in the GDNF/NSCs than NSCs. (e) Quantitative analysis of the percentages of NSCs and GDNF/NSCs differentiated into each type of neural cells (mean ± SD, *n* = 5). ^*∗*^Compared with NSCs group, *P* < 0.05. (f–k) Immunostaining showed, respectively, MAP2, GalC, and GFAP-positive cells from neurospheres. (f) MAP2^+^ cells from NSCs; (g) MAP2^+^ cells from GDNF/NSCs; (h) GalC^+^ cells from NSCs; (i) GalC^+^ cells from GDNF/NSCs; (j) GFAP^+^ cells from NSCs; (k) GFAP^+^ cells from GDNF/NSCs; (a–c) bar = 50 *μ*m; (f–k) bar = 75 *μ*m.

After 5–7 days of culture, neurospheres appeared (Figure 1(a)), which expressed the neuroepithelial cells specific markers nestin (Figure 1(b)). After they were infected by GDNF recombinant adenovirus for 2 days, the neurospheres displayed green fluorescence under the fluorescent microscope (Figure 1(c)) and strongly expressed GDNF mRNA (Figure 1(d)). To observe the differentiation, neurospheres were plated in coverslips with medium containing 10% FBS without bFGF, EGF, and N2 supplement for 7 days. The cell specific antibodies including MAP2, GFAP, and GalC were used to label differentiated cells. Compared to NSCs, the ratio of MAP2-positive neurons from GDNF/NSCs was significantly increased (*P* < 0.05), while the ratio of GFAP-positive astrocytes from GDNF/NSCs was significantly decreased (*P* < 0.05). Although the increased ratio of GalC-positive oligodendrocytes in GDNF/NSCs group was observed compared to NSCs, it did not reach the significant difference (*P* > 0.05) (Figures 1(e)–1(k)).

### 3.2. GDNF Accelerated the Recovery of NSCs on Clinical Symptoms

**Figure 2:**
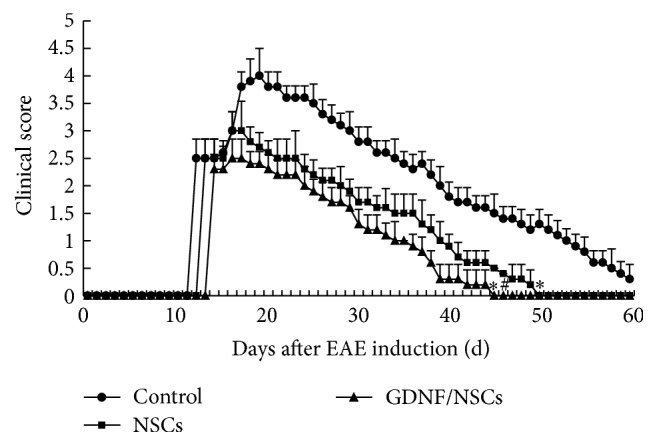
The improvement of clinical symptoms after transplantation (mean ± SD, *n* = 5). ^*∗*^Comparison with control group, *P* < 0.05; ^#^comparison with NSCs group, *P* < 0.05.

To evaluate the effects of NSCs on EAE rats, NSCs were transplanted into lateral ventricles before the onset of the disease (on day 10 after EAE induction). Disabilities appeared at 12 days after the initial EAE induction in the control rats, whereas cells transplantation delayed onset time, starting 1-2 days later. As shown in Figure 2, the rats receiving NSCs and those receiving GDNF/NSCs had reduced clinical symptoms compared to the control rats (*P* < 0.05). Moreover, the GDNF/NSCs rats had improved clinical outcomes relative to the NSCs group (*P* < 0.05). Although the control rats showed improved clinical scores over time, there were apparent deficits at the end of the follow-up period (60 days after EAE induction). The rats that received NSCs recovered their normal gait at 50 days after EAE induction, whereas the rats receiving GDNF/NSCs recovered at 45 days after EAE induction.

### 3.3. Attenuated Inflammation and More Myelin Sheath in GDNF/NSCs Compared to NSCs

**Figure 3:**
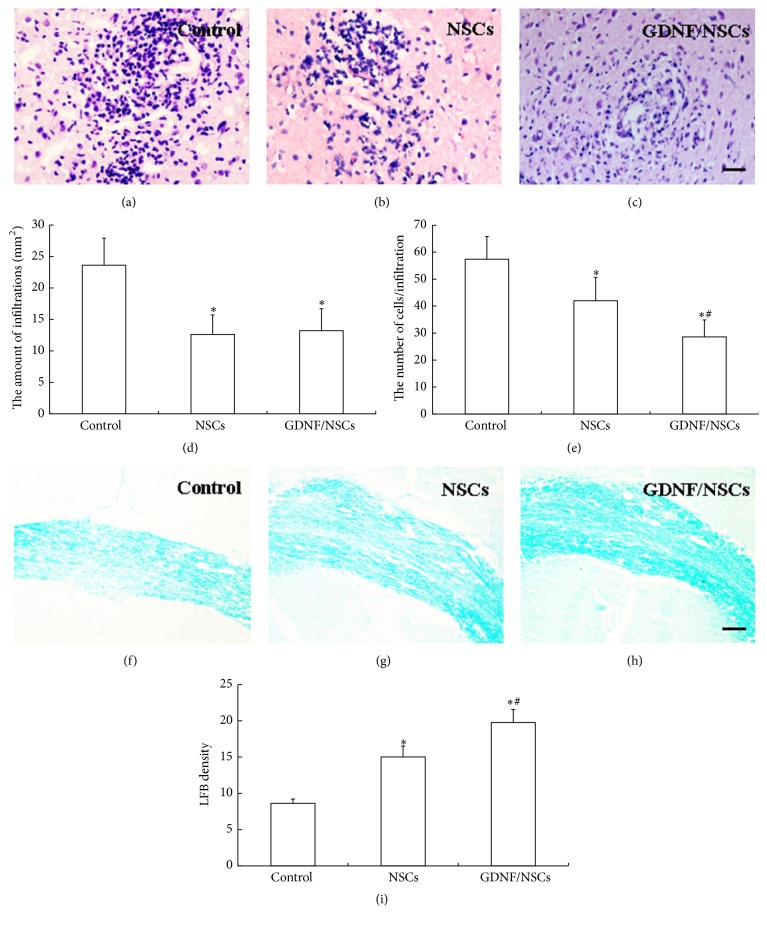
Attenuated inflammation and more myelin sheath in GDNF/NSCs compared to NSCs in EAE rats. (a–c) HE staining showed inflammatory infiltrates of the cerebral parenchyma in control, NSCs, and GDNF/NSCs groups, respectively. The control group showed much more infiltration with inflammatory cells. The transplanted groups displayed a reduction in the amount and size of the inflammatory infiltration, especially for the GDNF/NSC transplanted group. (d) Quantitative analysis of the amount of infiltration per mm^2^ (mean ± SD, *n* = 5). ^*∗*^Compared with control group, *P* < 0.05. (e) Quantitative analysis of the number of cells per infiltration (mean ± SD, *n* = 5). ^*∗*^Compared with control group, *P* < 0.05. ^#^Compared with NSCs group, *P* < 0.05. (f–h) LFB staining showed myelin sheath of the corpus callosum in control, NSCs, and GDNF/NSCs groups, respectively. Control group showed marked sparser and thinner myelin sheath compared with transplanted groups. In particular, GDNF/NSCs groups displayed more myelinated fibers than NSCs group. (i) Quantitative analysis of LFB density (mean ± SD, *n* = 5). ^*∗*^Compared with control group, *P* < 0.05; ^#^compared with NSCs group, *P* < 0.05. Bar = 100 *μ*m.

Inflammatory infiltrates and density of myelin sheath in brain of EAE rats were evaluated at 60 days after EAE induction. The majority of infiltrates were located in the corpus striatum adjacent to fibers, and fewer appeared in the cortex. The infiltrates in the corpus striatum were counted, and the results showed that the rats with saline showed more infiltrates than the rats with transplantation (*P* < 0.05) (Figures 3(a)–3(d)); moreover, fewer cells per infiltration were observed in rats with GDNF/NSCs compared with rats with NSCs (*P* < 0.05) (Figure 3(e)). The saline-injected rats showed sparse and thin myelin sheath (Figure 3(f)). In addition, myelin was observed by LFB staining. In our setting, phagocytic cells were also labeled by LFB staining. More myelin sheath indicated by stronger color intensity than control rats was observed in NSC transplantation, in particular GDNF/NSC transplantation (*P* < 0.05), although it may contain phagocytic cells (Figures 3(g)–3(i)).

### 3.4. GDNF Enhanced Survival and Neuron and Oligodendrocyte Differentiation of Transplanted NSCs

**Figure 4:**
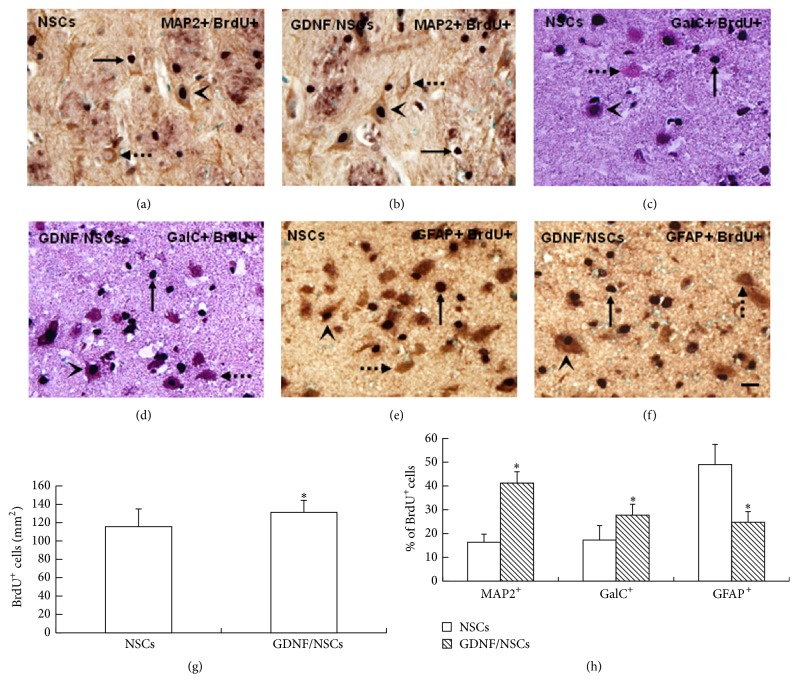
Double-immunohistochemical staining showed differentiation of transplanted NSCs or GDNF/NSCs in the corpus striatum. (a, b) BrdU^+^ cells (solid arrow), MAP2^+^ cells (dotted arrow), and MAP2^+^/BrdU^+^ cells (arrowhead) in NSCs group and GDNF/NSCs group, respectively. (c, d) BrdU^+^ positive cells (solid arrow), GalC^+^ cells (dotted arrow), and GalC^+^/BrdU^+^ cells (arrowhead) in NSCs group and GDNF/NSCs group, respectively. (e, f) BrdU^+^ cells (solid arrow), GFAP^+^ cells (dotted arrow), and GFAP^+^/BrdU^+^ cells (arrowhead) in NSCs group and GDNF/NSCs group, respectively. (g) Quantitative analysis of BrdU^+^ cells in NSCs and GDNF/NSCs groups (mean ± SD, *n* = 5). ^*∗*^Compared with NSCs group, *P* < 0.05. (h) Quantitative analysis of the ratio of transplanted NSCs and GDNF/NSCs differentiated into each type of neural cells (mean ± SD, *n* = 5). ^*∗*^Compared with NSCs group, *P* < 0.05. Bar = 25 *μ*m.

The transplanted cells were detected by BrdU-immunostaining in rats at 60 days after EAE induction. The BrdU^+^ cells located predominantly in the inflamed areas (Figures 4(a)–4(f)), and the density of BrdU^+^ cells per mm^2^ in rats with GDNF/NSCs was significantly increased compared to rats with NSCs (*P* < 0.05) (Figure 4(g)). The MAP2^+^/BrdU^+^ cells and GalC^+^/BrdU^+^ cells in rats with GDNF/NSCs were apparently higher compared to rats with NSCs (*P* < 0.05); in contrast, the GFAP^+^/BrdU^+^ cells in rats with GDNF/NSCs were decreased compared to rats with NSCs (*P* < 0.05) (Figure 4(h)), which suggested that GDNF/NSCs differentiated into more neurons and oligodendrocytes but fewer astrocytes compared to NSCs in the EAE-induced rats.

### 3.5. GDNF Increased the Expression of mRNA for Oligodendrocyte Lineage Cells

**Figure 5:**
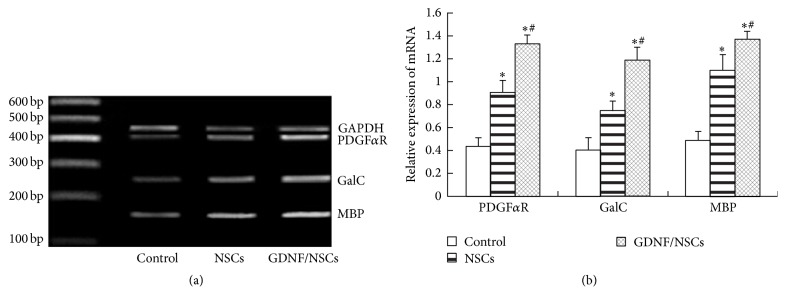
GDNF increased expression of mRNA for oligodendrocyte lineage cells. (a) mRNA expression of PDGF*α*R, GalC, and MBP in the cerebral lesion areas in each group. (b) Relative expression of PDGF*α*R, GalC, and MBP mRNA following transplantation (PDGF*α*R/GAPDH, GalC/GAPDH, and MBP/GAPDH) (mean ± SD, *n* = 5). ^*∗*^Compared with control group, *P* < 0.05; ^#^compared with NSCs group, *P* < 0.05.

The PDGF*α*R, the marker of oligodendrocyte progenitor cells, GalC, the marker of immature and mature oligodendrocytes, and MBP, the markers of myelinating oligodendrocytes, were used to evaluate oligodendrocyte lineage cells after transplantation. NSCs and GDNF/NSCs transplantation significantly increased mRNA expressions of PDGF*α*R, GalC, and MBP compared with control group (*P* < 0.05), and GDNF/NSCs transplantation appeared more efficient than NSCs (*P* < 0.05) (Figures 5(a) and 5(b)). These results showed that oligodendrogenesis and remyelination are significantly increased following NSCs transplantation, especially GDNF/NSCs transplantation.

## 4. Discussion

Our study showed that grafting NSCs and GDNF/NSCs into rats subjected to EAE significantly improved function compared with control group, and GDNF/NSCs group showed better efficacy. More myelin sheath and fewer inflammatory infiltration were observed in GDNF/NSCs group. More BrdU^+^ GDNF/NSCs were observed compared to BrdU^+^ NSCs, and more MAP2 and GalC-positive cells from GDNF/NSCs were detected compared with transplanted NSCs. Moreover, the increased expression of mRNA for PDGF*α*R, GalC, and MBP in brains was observed in GDNF/NSCs group compared to NSCs group.

Previous studies have reported that NSCs transplantation could reduce EAE-induced inflammation of CNS and promote clinical improvement [15–17]. When injecting subcutaneously, NSCs migrated into lymph nodes of EAE mice where they hampered the activation of myeloid dendritic cells and steadily restrained the expansion of encephalitogenic T cells, therefore reducing immune cell mobilization from the periphery [17, 18]. Transplanted systemically, NSCs entered perivascular CNS areas and subsequently induced apoptosis of blood-borne CNS-infiltrating encephalitogenic T cells [7, 15, 19]. Previous studies have shown that NSCs transplantation ameliorated the clinical symptoms and reduced tissue injury after EAE, which was related to the reduction in the number of perivascular infiltrates and of brain encephalitogenic T cells [20]. In this study, we showed a more significant reduction in the amount and size of inflammatory cell infiltration in GDNF/NSCs group compared with NSCs group. These results indicate that GDNF/NSCs more effectively reduce infiltrating inflammatory cells, which may result in greater therapeutic efficacy than NSCs on EAE, whereas the limitation in the present study is that control NSCs should be infected by empty virus vector expressing GFP alone because dividing cells may dilute out the BrdU label.

An inflammatory environment inevitably results in axonal degeneration and demyelination, which is correlated with chronic disability and brain atrophy in advanced MS [21]. Previous studies have suggested that transplanted NSCs generated new neurons and oligodendrocyte lineage cells to replace lost or degenerative cells, markedly promoting axonal regeneration and decreasing the extent of demyelination [16, 22]. The present results showed that more new neurons and oligodendrocytes were generated from transplanted GDNF/NSCs than that from transplanted NSCs, at least partially providing the possibility of remyelination and may result in better clinical recovery. Moreover, under pathological environments in the CNS, transplanted NSCs differentiate into astrocytes, which potentially cause reactive gliosis [23, 24] and hamper endogenous axonal remyelination [7, 13]. Here we showed that GDNF/NSCs generated less astrocytes than NSCs in EAE brain, which may be conducive to remyelination.

PDGF*α*R, GalC, and MBP, the markers of oligodendrocyte lineage cells, were strongly diminished at the chronic phase of EAE [21, 25, 26]. In this study, cells transplantation increased the expression of mRNA for PDGF*α*R, GalC, and MBP in the lesion areas in the brain, with higher expressions in the GDNF/NSCs group compared with the NSCs group. This suggests that a large number of oligodendrocyte lineage cells are generated after grafting, especially GDNF/NSCs. Therefore, rats with GDNF/NSCs grafting may possess more extent of remyelination than rats with NSCs for more myelin sheath by LFB staining was observed although the possibility that phagocytic cells were contained in these regions could not be excluded.

In summary, we showed here that GDNF/NSCs grafting significantly promotes functional recovery in EAE rats, reducing brain inflammatory infiltration, improving density of myelin, increasing repopulation of neurons and oligodendrocyte lineage cells, and decreasing the astrocyte differentiation of NSCs. These effects suggest that GDNF augments therapeutic efficiency of NSCs-based therapy on chronic EAE, providing a more promising approach to therapy EAE.
